# NaAlTi_3_O_8_, A Novel Anode Material for Sodium Ion Battery

**DOI:** 10.1038/s41598-017-00202-y

**Published:** 2017-03-13

**Authors:** Xuetian Ma, Ke An, Jianming Bai, Hailong Chen

**Affiliations:** 1 The Woodruff School of Mechanical Engineering, Georgia Institute of Technology, Atlanta, GA 30332 USA; 2 Chemical and Engineering Materials Division, Oak Ridge National Laboratory, Oak Ridge, TN 37831 USA; 3 National Synchrotron Light Source II, Brookhaven National Laboratory, Upton, NY 11973 USA

## Abstract

Sodium ion batteries are being considered as an alternative to lithium ion batteries in large-scale energy storage applications owing to the low cost. A novel titanate compound, NaAlTi_3_O_8_, was successfully synthesized and tested as a promising anode material for sodium ion batteries. Powder X-ray Diffraction (XRD) and refinement were used to analyze the crystal structure. Electrochemical cycling tests under a C/10 rate between 0.01 - 2.5 V showed that ~83 mAh/g capacity could be achieved in the second cycle, with ~75% of which retained after 100 cycles, which corresponds to 0.75 Na^+^ insertion and extraction. The influence of synthesis conditions on electrochemical performances was investigated and discussed. NaAlTi_3_O_8_ not only presents a new anode material with low average voltage of ~0.5 V, but also provides a new type of intercalation anode with a crystal structure that differentiates from the anodes that have been reported.

## Introduction

Lithium Ion Battery (LIB) has been widely used in mobile electronics such as smart phones and laptop computers in the last two decades^1,2^. However, for applications that require large-scale electricity storage, such as electrical grid storage, the high cost of LIBs has become a major barrier^3^. The high cost of LIBs is partially due to the use of relatively precious metals, such as lithium, cobalt and nickel. This cost of raw materials will not likely be reduced with the optimization of manufacturing process and the growth of production^4^. Therefore, alternative energy storage technologies with lower cost, higher energy densities^5^, and high power densities^6–8^ are being actively considered. Sodium Ion Battery (SIB) is one of the emerging energy storage technologies with high promise to be manufactured at lower cost in the future^9^. Two major factors can significantly contribute to the lower cost of SIBs, compared with LIBs. First is that the natural abundance of sodium in the earth’s crust is 1000 times higher than that of lithium (23,600 ppm vs. 20 ppm)^10^ and the distribution of sodium resources is not as geologically limited as that for lithium. Second is that in SIBs, cheaper and lighter aluminum foil can be used as the current collector on the anode side^11^, in comparison with the expensive and heavy copper foil that must be used for anode in LIBs^12,13^, which will both lower the cost and help in improving the energy density.

SIB resembles LIB in structure and functioning mechanisms. It contains cathode, anode, electrolyte and porous separator. Na ions shuttle between cathode and anode in electrochemical cycling to form a “rocking chair” type of rechargeable battery^14^. Different from the rather mature LIB technology, currently, many key components of SIBs, including cathode, anode, and electrolyte, still require significant improvements in many aspects to enable a commercial competition with LIBs^15^. On cathode side, many compounds with capacities that are comparable to lithium cathodes have recently been reported, such as P2-Na_0.66_(Fe_0.5_Mn_0.5_)O_2_^16^. However, on the anode side, much less types of materials are being considered. Unlike lithium ion, sodium ion cannot intercalate into graphite, the most commonly used anode for LIBs, due to the unfavorable thermodynamics^17^. Among the up-to-date work on anode materials, carbonaceous materials, alloys, and oxides are of major interests. Among carbonaceous anodes, hard carbon exhibits a high reversible capacity ~250 mAh/g with good retention^18^, and therefore is considered as the “first generation anode” of SIBs^4^. However, the possible Na plating issue associated with the very low discharge plateau with Na/Na^+^ at 0 - 0.1 V remains to be addressed. Alloys such as Sn and Sb are attractive because of their very high specific capacity, which is ~500 - 600 mAh/g^19^. But similar to what is well-known for Si and alloys in LIBs^20^, the issue related to this type of anode is the large volume change during Na ion insertion and extraction, which may lead to cracking of particles, and the resulted capacity fading. The intercalation-type anode has the advantage of low volume change in cycling^21,22^. However, not many crystal structures and compounds have been reported. Sodium titanates are particularly interesting in that many of them have low intercalation potential and very low or even zero volume change at the end of charge and discharge states^23^. Na ion can be inserted into Li_4_Ti_5_O_12_ via a three-phase pathway^24^ and a ~145 mAh/g reversible capacity can be achieved at about 1 V vs. Na/Na^+^. A P2-type layer metal oxide, Na_0.66_[Li_0.22_Ti_0.78_]O_2_^19^, claimed as a “zero-strain” anode material, exhibits only ~0.77% volume change during sodium insertion/extraction and delivers ~116 mAh/g reversible capacity at ~0.75 V. Na_2_Ti_3_O_7_ and Na_2_Ti_6_O_13_ are two sodium titanates being reported to have diverse structural and electrochemical properties. Na_2_Ti_3_O_7_ has very low average voltage, at 0.5 V vs. Na/Na^+^ and it exhibits more than 200 mAh/g initial discharge capacity and ~177 mAh/g reversible discharge capacity^25^. Na_2_Ti_6_O_13_ shows lower capacity of ~45 mAh/g, corresponding to 1 Na^+^ insertion per formula. However, it performs well at high rates (up to 30 C) and shows very long cycle life (>5000 cycles) and good retention of 85% of initial capacity after 5000 cycles under 20 C^26^. The above mentioned compounds have shown the promise of titanates and large chemical space for further exploration and material design.

In this work, we report the synthesis and electrochemical testing of another intercalation titanate, NaAlTi_3_O_8_, which is similar to Na_2_Ti_3_O_7_ in theoretical capacity but with a different crystal structure. If all the three Ti atoms can be reduced from 4^+^ to 3^+^, the compound provides a theoretical capacity of 250 mAh/g, which is slightly lower than that of Na_2_Ti_3_O_7_, which is 266 mAh/g. In addition, cheaper element Al is used here instead of all Ti, which can further lower the cost of the material. The intercalation of Na into this type of crystal structure has never been studied and reported before. A systematical study can reveal the insights on the intercalation mechanism and shed light on design of new materials with high capacity and excellent stability.

## Results and Discussion

**Figure 1:**
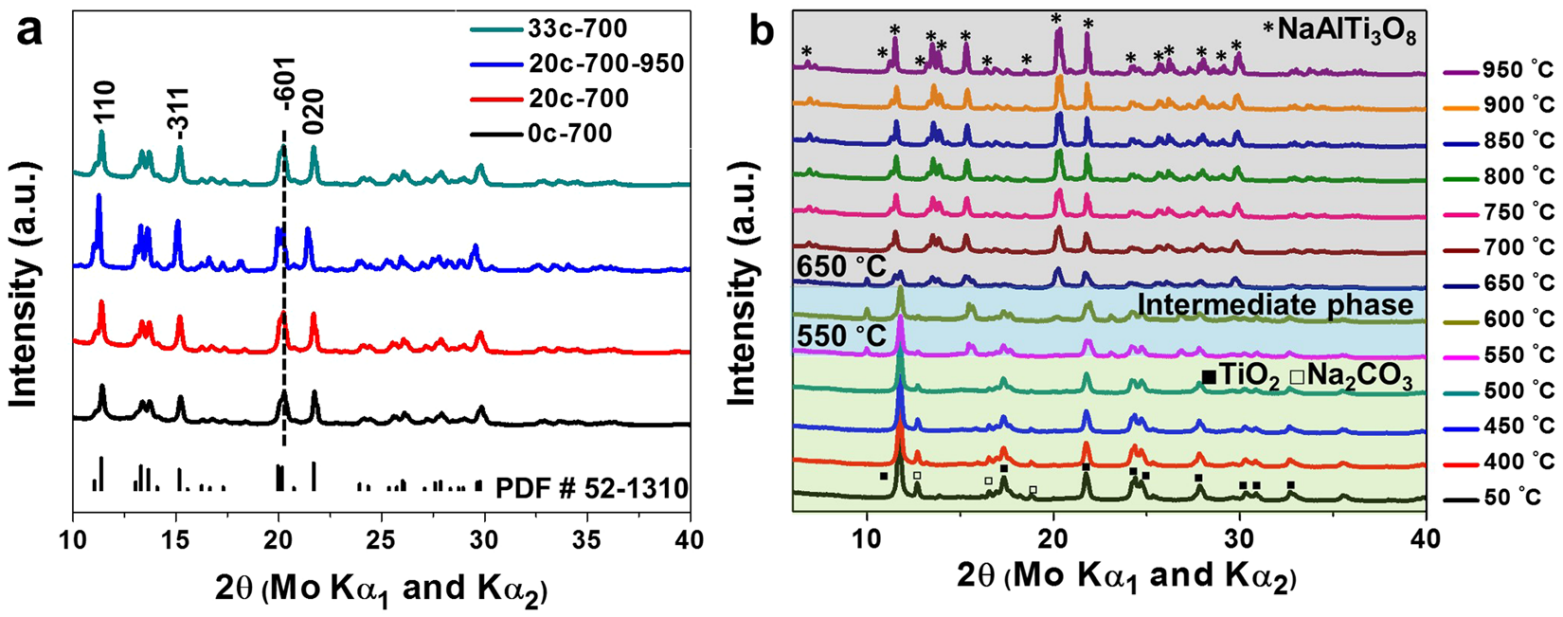
(**a**) XRD patterns of 0 C, 20 C, 33 C samples under 700 °C -10 h synthesis condition and 20 C sample under 700 °C for 10 h and 950 °C for another 2 h; PDF #52-1310 is labeled with the bars at the bottom. (**b**) *In situ* XRD of 0 C sample heated from 50 °C to 950 °C.

NaAlTi_3_O_8_ is a known compound that has been investigated for its thermal expansion properties. But to the best of our knowledge, it has not been investigated for the ion-storage properties in either Li-ion or Na-ion batteries. NaAlTi_3_O_8_ samples were synthesized through solid-state-reaction using stoichiometric amount of Na_2_CO_3_, Al_2_O_3_, and anatase-TiO_2_. Considering that many of the Ti^4+^ titanates have low electronic conductivity, sucrose is mixed into the starting material to form carbon coating as widely being used for low conductive materials such as LiFePO_4_^23^. Samples with 0 wt%, 20 wt% and 33 wt% sucrose of the starting materials are denoted as 0 C, 22 C, and 33 C hereafter, respectively. The mixture was ball-milled at 400 rpm for 1 hour and pressed into pellets. The pellets were calcined under argon flow at 700 °C for 10 h or 700 °C - 10 h followed by 950 °C - 2 h. The pellets of as-synthesized samples were ground into powder for X-ray Diffraction (XRD) analysis. All the obtained samples, 0 C, 20 C, and 33 C that are sintered at 700 °C for 10 h are confirmed to have the same pattern with PDF#52-1310 Na_2_Al_2_Ti_6_O_16_, as shown in Fig. 1(a). The 0 C sample has a white color, as expected for most Ti^4+^ oxides, indicating an intrinsic insulating property that is not favorable for electrochemical cycling. The decomposition of sucrose at 700 °C forms uniform carbon coating on the surface of NaAlTi_3_O_8_ particles and makes 20 C and 33 C samples very deep-dark color. On the other hand, the carbon coating does not change the crystallinity or crystalline size of the samples, evident by the 0 C, 20 C, and 33 C samples showing similar width of XRD peaks. Figure 1(a) also shows the comparison of 20 C sample at different calcination conditions, namely 700 °C for 10 h and 700 °C for 10 h followed by 950 °C for 2 h. Both samples yield pure phase of NaAlTi_3_O_8_, but the sharper peaks in the 700 °C - 950 °C sample indicate better crystallinity and larger particle size under higher temperature. In addition, the color of the 700 °C - 950 °C sample is less dark compared to the 700 °C sample, likely due to the loss of carbon at higher temperature.

To investigate the formation of the NaAlTi_3_O_8_ phase in the solid-state synthesis, *in situ* XRD for synthesis was performed with using an Anton Parr furnace chamber mounted on the diffractometer. The pellet of starting materials was heated in the chamber from room temperature (r.t.) to 950 °C. The XRD patterns were taken at 50 °C and every 50 °C from 400 °C to 950 °C. The scanning time was one hour, while the temperature was held constant during the scan. In the 50 °C pattern, the peaks can be indexed as the starting materials, Na_2_CO_3_ and anatase-TiO_2_. Al_2_O_3_ peaks are invisible due to the small amount and the amorphous nature. It can be seen that the reaction starts as early as 550 °C and possibly an unknown intermediate phase forms at 550 °C, evident by the appearance of peaks at 10 and 15 degrees. The NaAlTi_3_O_8_ phase forms at around 700 °C and stays along at higher temperatures. The *in situ* XRD shows that the synthesis reaction is rather straightforward and no additional polymorphs formation or phase transitions are present. Based on the *in situ* XRD result, the synthesis condition to form samples with good crystallinity is determined to be no lower than 700 °C.

**Figure 2:**
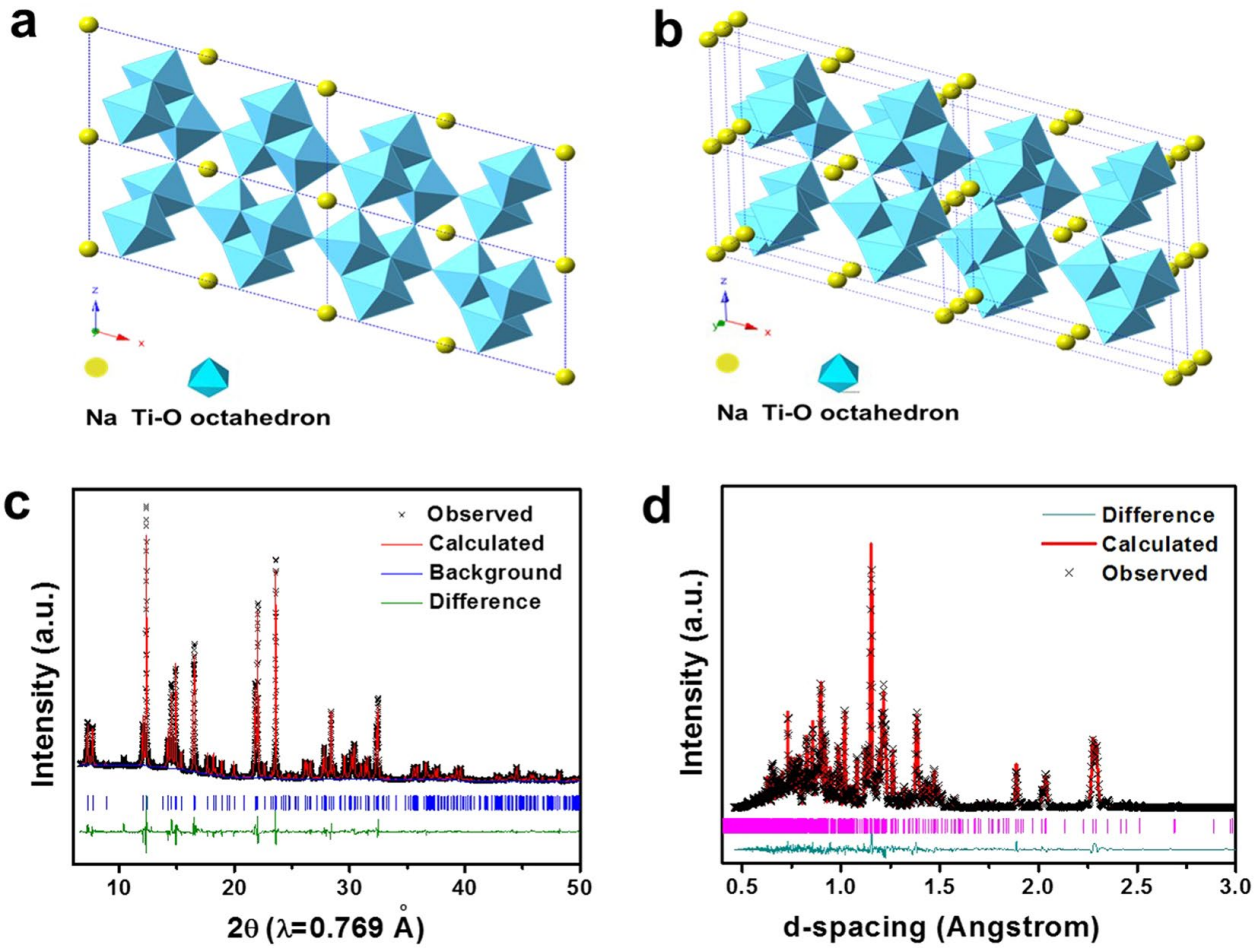
(**a**) Projection of NaAlTi_3_O_8_ along [010] direction. (**b**) 3D structure tilted from [010] direction. (**c**) Rietveld refinement of NaAlTi_3_O_8_ using synchrotron XRD. (**d**) Rietveld refinement of NaAlTi_3_O_8_ using neutron scattering.

Figure 2(a) depicts the projection of NaAlTi_3_O_8_ crystal structure along *b* axis and (b) shows the 3D structure tilted from *b* axis. The compound has a three-dimensional tunnel structure composed of corrugated layers of edge-sharing TiO_6_ octahedrons. Al is randomly mixed with Ti at the octahedral sites. These octahedral slabs are jointed by corners in *c* axis and share edges along *a* axis, composing the tunnel framework. High resolution synchrotron X-ray diffraction patterns of the samples were collected at beam line X14A at the National Synchrotron Light Source (NSLS) at Brookhaven National Laboratory (BNL) and beam line 17-BM at the Advanced Photon Source (APS) at Argonne National Laboratory (ANL) to detect the structural feature of this compound. The crystal structure was refined by Rietveld method using diffraction pattern collected at X14A at NSLS, as shown in Fig. 2(c). A monoclinic structure with C2/m space group is used and the lattice parameters are summarized in Table 1. The Rietveld refinement confirms that Al shares the same crystallographic site 4i as Ti and no obvious ordering is observed. Sodium takes the 2a site. To further confirm the coordination of oxygen and sodium, neutron scattering was performed at the VULCAN beam line at the Spallation Neutron Source (SNS) at Oak Ridge National Laboratory (ORNL). Refinement result of the neutron scattering data (Fig. 2d) agrees with the refinement of synchrotron XRD, as provided in Table 1.

**Table 1:** Rietveld refinement results of crystal parameter for NaAlTi_3_O_8_ data taken from synchrotron X-ray diffraction and neutron scattering.

	*a/Å*	*b/Å*	*c/Å*	*α/°*	*β/°*	*γ/°*	*wRp*	*rp*	*Space Group*
**X-ray**	12.10(8)	3.77(3)	6.40(8)	90	107.61(7)	90	5.65%	4.07%	C2/m
**Neutron**	12.11(3)	3.77(4)	6.41(3)	90	107.61(1)	90	3.73%	2.78%	C2/m

**Figure 3:**
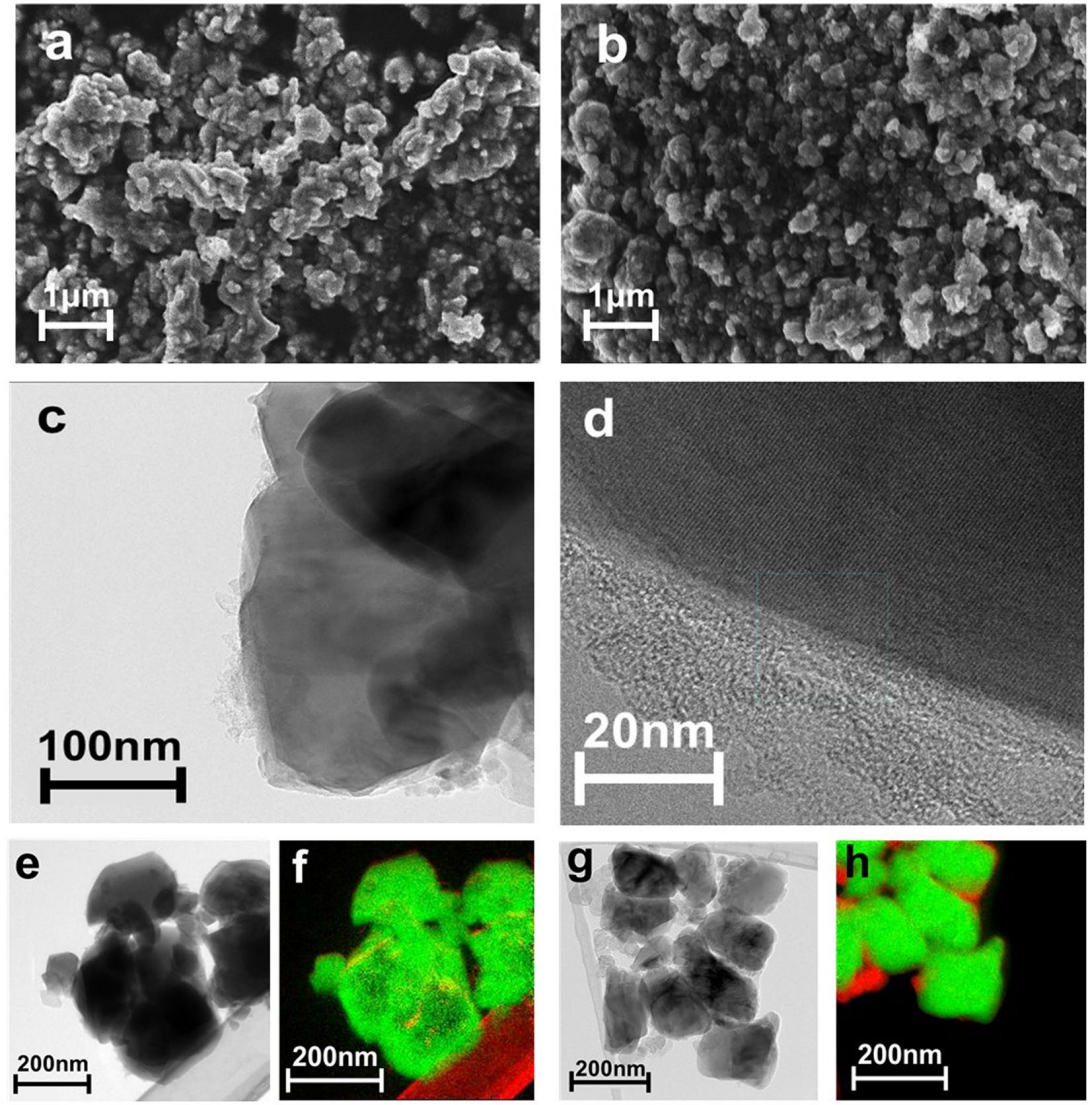
(**a**) SEM image of 20 C sample under 700 °C for 10 h and 950 °C for 2 h with no ball-milling treatment. (**b**) SEM image of 20 C sample under 700 °C for 10 h and 950 °C for 2 h, followed by ball-milling with carbon black at 600 rpm for 1 hour. (**c**) and (**d**) TEM images of sample in (**a**). (**e**), (**f**) EELS images of sample in (**a**). (**g**), (**h**) EELS images of sample in (**b**).

Samples were ball-milled with carbon black (CB) with a ratio of 85:15 before being used for electrochemical testing. Scanning Electron Microscope (SEM) and Transmission Electron Microscope (TEM) were applied to investigate and compare the particle morphology under different synthesis conditions. Energy Dispersive X-ray Spectroscopy (EDS) was used to visualize the elemental distribution of 20 C-NaAlTi_3_O_8_ without ball-milling with carbon black. It was found that all elements distributed uniformly across particles, as shown in Supplementary Fig. S1. Electron Energy-Loss Spectroscopy (EELS) was applied to visualize the distribution of carbon under different synthesis conditions. Figure 3(a) and (b) show SEM images of NaAlTi_3_O_8_ sample under the following conditions: (a) 20 C sample synthesized at 700 °C -10 h followed by 950 °C - 2 h; (b) 20 C sample synthesized at 700 °C for 10 h followed by 950 °C for 2 h, after which it was ball-milled with CB at 600 rpm for 1 hour. The magnification is ~38 k and the voltage is 5 kV. In Fig. 3(a) the particles are of irregular shape and the size is ~200 - 500 nm, with slight agglomeration. In Fig. 3(b), after ball-milling, the particle morphology is more homogeneous. The particle size decreases to ~100 - 200 nm, with more obvious agglomeration. Figure 3(c) and (d) show the TEM images of 20 C sample synthesized without ball-milling with CB. The sucrose resulted in a uniform thin coating layer on the surface of particles, with a thickness of ~20 - 30 nm, which would be helpful in improving the conductivity of the material during electrochemical cycling. Figure 3(e - g) and (h) show the EELS images of 20 C samples without ball-milling (e, f) and with ball-milling (g, h). The green regions show the distribution of Ti element, while the red regions show that of C element. For the 20 C sample without ball-milling, the carbon mostly exists as the coating layer on the particles, in accordance with Fig. 3(c) and (d). After ball-milling with CB, the particle size decreases and particles become more uniform, with carbon existing mostly in the space between particles, which can be easily seen from Fig. 3(h). Those carbon particles help increase the overall conductivity of NaAlTi_3_O_8_ and the reduced size may shorten Na ion’s diffusion path and improve the electrochemical performance.

All samples were made into electrode films with conventional doctor-blade method and tested in coin-cell type half cells with using pure sodium metal foil as the anode. Sample without carbon coating, as provided in Supplementary Fig. S2, shows low capacity of about 98 mAh/g for the 1^st^ cycle and 48 mAh/g of discharge capacity for the 2^nd^ cycle under the rate of C/5, likely due to the poor electronic conductivity that is common for Ti^4+^ oxides^27^. To reduce the barrier causing this poor electronic conductivity so as to investigate the true sodium storage and conducting properties of this compound, carbon coating in synthesis and carbon mixing with ball-milling were used to improve the electronic conductivity, as has been done with LiFePO_4_^28^. For 20 C and 33 C samples, in which 20 and 33% weight percent of sucrose were used as the carbon source in synthesis, Inductive Couple Plasma (ICP) spectroscopy was used to measure the carbon content in the final products. The 20 C sample contains 5% carbon and 33 C sample contains 8% carbon. The electrochemical performance of these two samples were very similar, implying that 5% carbon is sufficient to provide good electron conduction and additional carbon is excessive. For the reason hereby we only focus on the electrochemical tests results from the 20 C sample with ball-milling with carbon black.

**Figure 4:**
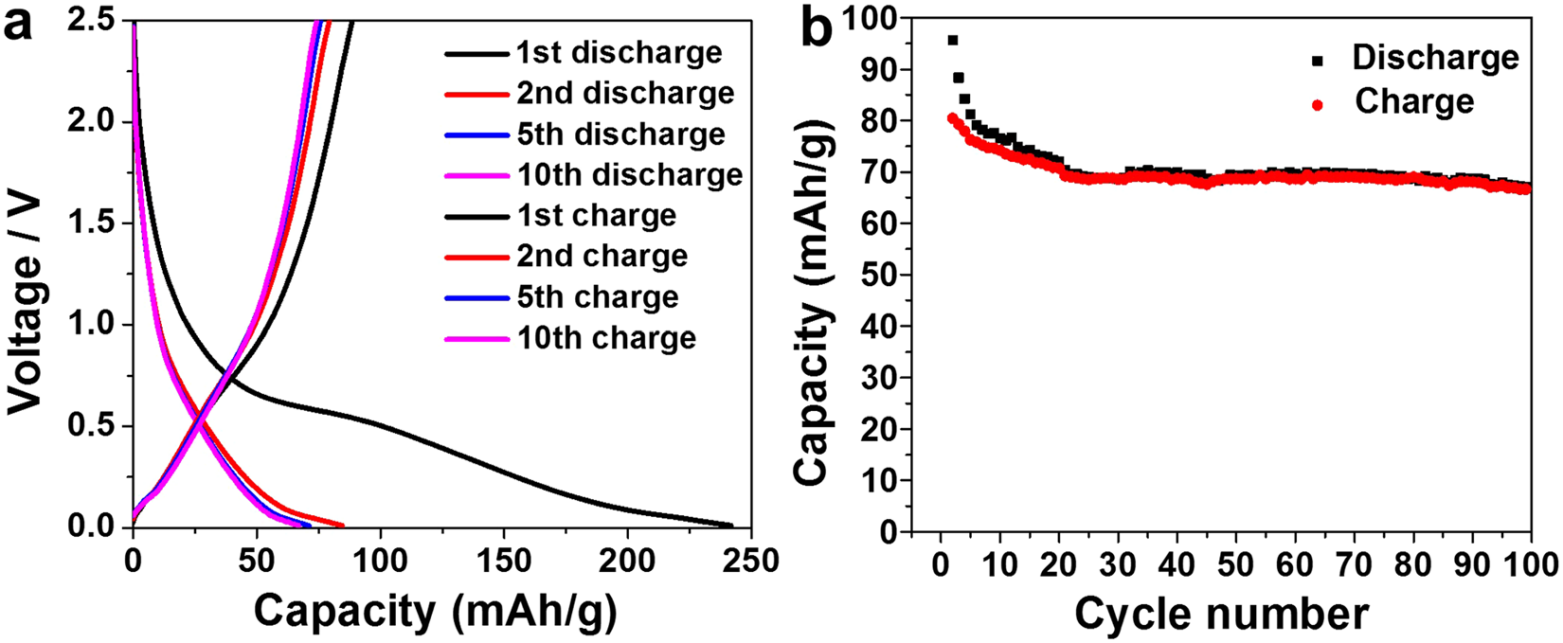
(**a**) 1^st^, 2^nd^, 5^th^, and 10^th^ discharge and charge profiles at the rate of C/10 of 20 C sample under 700 °C for 10 h and 950 °C for another 2 h. (**b**) Discharge and charge capacity from the 2^nd^ to the 100^th^ cycle.

Figure 4(a) shows the 1^st^, 2^nd^, 5^th^, and 10^th^ discharge and charge voltage profiles of the 700 - 950 °C calcined 20 C NaAlTi_3_O_8_ sample, at a C/10 rate between 0.01 and 2.5 V. The average discharge voltage is ~0.5 V, which is close to what was observed for Na_2_Ti_3_O_7_^23^. The low voltage is good for anode materials. In addition, this voltage is high enough above 0 V, therefore will be effective to suppress sodium plating during discharge in full cells^29^. The voltage curves in both charge and discharge are slopping and no obvious plateaus are observed, implying that this material may undergo a solid solution-like phase evolution pathway. The first discharge capacity is 225 mAh/g, roughly corresponding to 3 Na^+^ insertion while the first charge capacity is 80 mAh/g, corresponding to 1 Na^+^ extraction. However, part of this high 1^st^ discharge capacity should be due to the SEI formation and the capacity from the carbonaceous components in the composite electrode. In the second cycle, the discharge capacity quickly drops to ~83 mAh/g and stabilizes around 62 mAh/g in the following cycles. The significant large irreversibility of capacity is similar to what was observed in Na_2_Ti_3_O_7_ again^23^, likely due to an unstable end-of-discharge structure with too many Na ions being inserted into the lattice, which results in sliding and distorting of the Ti-O slabs. The stabilized capacity for both discharging and charging processes is 62 mAh/g, corresponding to 0.75 Na ion intercalation/de-intercalation (theoretical capacity of 1 Na insertion is 83 mAh/g). However, the capacity retention after the second cycle to over 100 cycles is very good. About 75% capacity of the second discharge sustains in the 100^th^ cycle. It is worth noting that the capacity values discussed above are the numbers that have subtracted the capacity contribution from carbonaceous components, including coated carbon, ball-milling mixed carbon, and additional carbon. The capacity of carbon, as shown in Supplementary Fig. S3, was estimated by making a pure carbon black film and cycled it under exactly the same conditions and calculated according to the weight percentage of carbon in each sample. In this way, it is also confirmed that the capacity shown in Fig. 4 is indeed from the active material.

**Figure 5:**
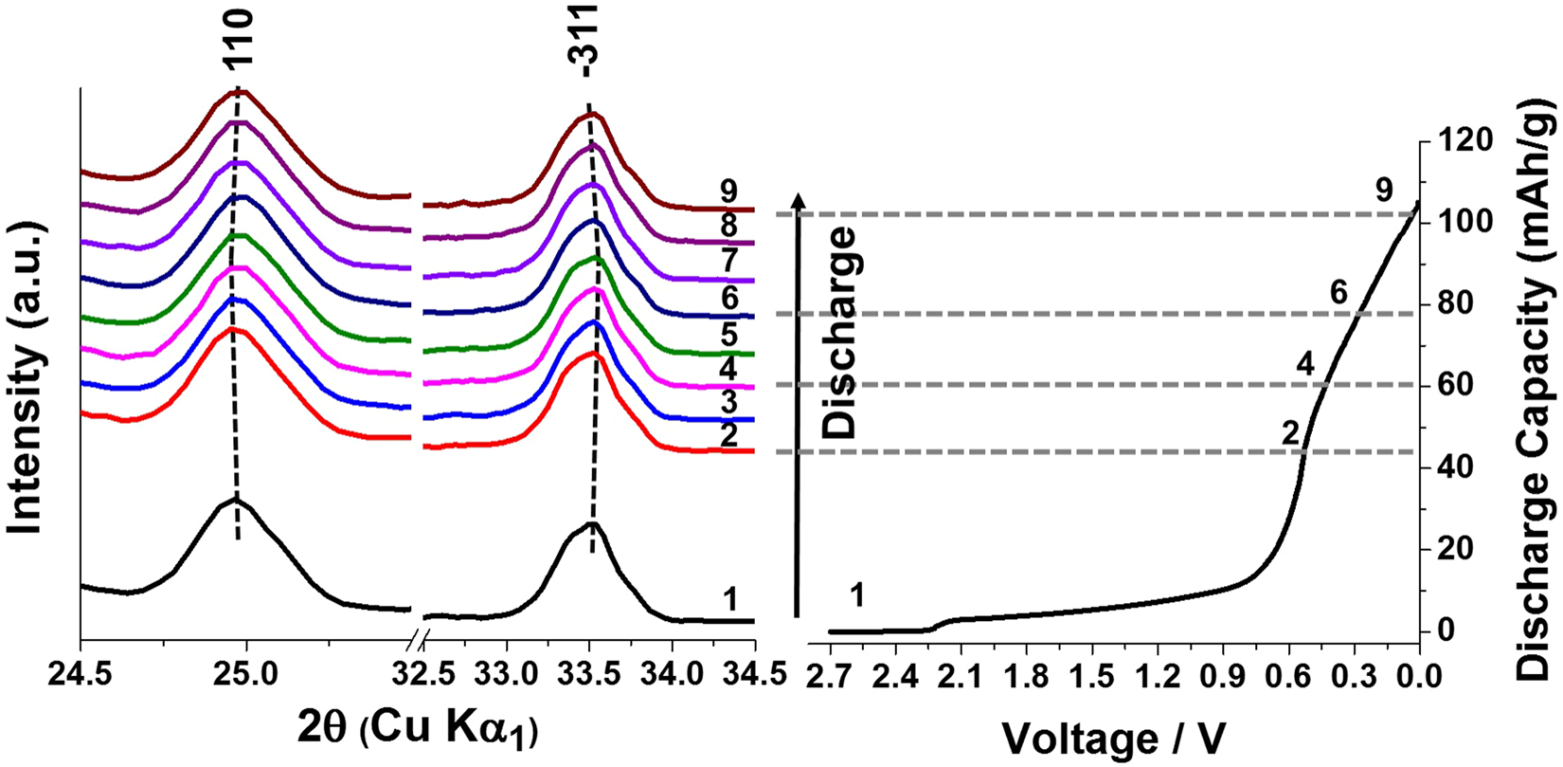
*In situ* XRD patterns during the first discharge at a rate of C/40, within the range of 2.71 to 0.01 V. The peaks shown on the left are (110) and the overlapping peaks of (−311) and (310), respectively.

To understand the structure evolution in the electrochemical cycling, *in situ* XRD was conducted with using an in-house *in situ* battery XRD system. The *in situ* cell was assembled as shown in Supplementary Fig. S4. The battery was discharged from an open circuit voltage (OCV) of 2.71 V with a discharge rate of C/40. Each XRD scan took 2 hours. The spectra 1 - 9 correspond to discharge voltage at 2.71 V, 0.553 - 0.53 V, 0.529 - 0.493 V, 0.491 - 0.437 V, 0.436 - 0.376 V, 0.376 - 0.302 V, 0.301 - 0.23 V, 0.228 - 0.155 V, and 0.137 - 0.054 V, respectively. The spectra were converted to Cu wavelength to better visualize the lower-angle peak positions. The positions of the peaks were corrected by carefully aligning the peaks from the beryllium window and aluminum current collector, in which way the peak shifting caused by the cell breathing effect can be corrected, and the accurate peak positions for each spectrum can be extracted. It is found that the changes in XRD patterns are subtle as many peaks showing anisotropic but slight shifting. For example, in Fig. 5, peak (110) slightly shifts to lower 2 - theta angle and peak (−311) slightly shifts to higher 2 - theta angle. The small yet clear shifting indicates a solid solution-like phase evolution pathway and very little volume change during discharge. This indicates that the Na ion is inserted into the structure through an intercalation mechanism during discharge. The very little volume change, though, is not uncommon for titanates. Quite a few compounds, such as P2-Na_0.66_Li_0.22_Ti_0.78_O_2_^19^ and Li_4_Ti_5_O_12_^30^, also exhibit very little volume change, and are recognized as zero-strain materials. No new phase forms during the discharge range that is implemented in the *in situ* cell. Note that due to a slightly higher resistance and over-potential in the *in situ* cell than that in the coin cells, the capacity that is achieved in the *in situ* cell in the first cycle is much smaller than that is observed in the coin cells.

Ex situ XRD was done to investigate the phase change after cycling of 20 C sample under C/10 for 20 cycles, which shows very little change in major peaks, in accordance with the *in situ* observation (see Supplementary Fig. S6). The result further proves the stability of the phase during cycling. SEM images of the before- and after- cycling samples show little difference in terms of morphology (see Supplementary Fig. S5). Both XRD and SEM results of the cycled samples suggest a fairly good stability of NaAlTi_3_O_8_ in electrochemical cycling.

**Figure 6:**
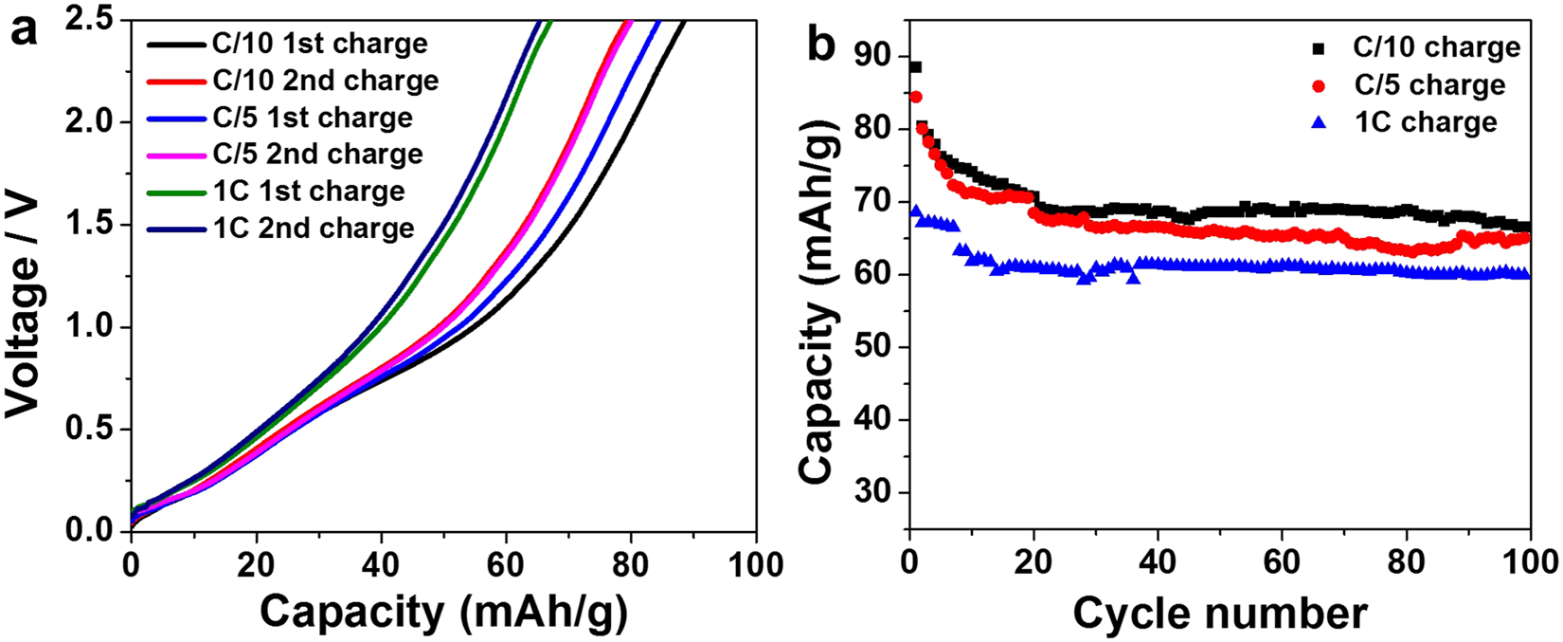
(**a**) 1^st^ and 2^nd^ charge profiles at C/10, C/5, and 1 C rates. (**b**) Charge capacities at C/10, C/5, and 1 C rates between 0.01 - 2.5 V.

Rate tests were conducted using 700 - 950 °C 20 C sample under C/10, C/5, and 1 C between 0.01 and 2.5 V. The rate is calculated with using 2 Na insertion as the theoretical limit, corresponding to 166 mAh/g. Figure 6(a) shows the 1^st^ and 2^nd^ charge profiles for each rate. The charge capacities from C/5 cycling are only slightly lower than those from C/10 cycling. The capacities at 1 C are about 20% lower than those from C/5 and C/10, indicating a moderate rate capability of this material. Figure 6(b) shows the long-term cycling profiles for the three rates up to 100 cycles. The long cycling capability is good for both higher and lower rates, though less capacity is delivered at 1 C rate.

High energy ball-milling is employed to obtain higher capacity for NaAlTi_3_O_8_. The rotation speed of ball-milling is critical in this case. Samples ball-milled at 600 rpm deliver higher capacity than those ball-milled at 500 rpm and not ball-milled, suggesting smaller particle size and more uniform morphology facilitate the diffusion of Na^+^.

From the investigation on NaAlTi_3_O_8_ samples, it is clear that this compound can be cycled in sodium ion batteries as an intercalation anode with a reasonable capacity of about one Na^+^ insertion and extraction. The initial discharge capacity of the compound is comparable to Na_2_Ti_3_O_7_, a similar intercalation titanate, however, with a more significant capacity drop in the following cycles. It is not clear yet at this point why the capacity is so much lower than the chemical theoretical value allowed by the Ti^4+^/Ti^3+^ redox couple. It is suggested that in Na_2_Ti_3_O_7_, the fading capacity is due to the unstable end-of-discharge structure, which is likely to be Na_4_Ti_3_O_7_^31^. While with NaAlTi_3_O_8_, no obvious difference between the structure of the pristine and discharged material is revealed by the *in situ* XRD. It may undergo a very subtle structural rearrangement that calls for further study with high resolution *in situ* synchrotron XRD or *in situ* neutron scattering study which is more sensitive to the arrangement of light elements such as O and Na. In addition, the poor electronic conductivity of this material requires carbon coating to make it function with a reasonable rate in the cell. The long term cycling performance of this material is indeed very good, plausibly benefiting from both the small volume change in cycling and the solid solution-like phase evolution pathway. On the other hand, this compound presents a new type of crystal structure that can undergo Na ion insertion and extraction with very little volume change, which can be beneficial for long-term cycling of full cells^27^. This is another titanate material with very low average voltage, comparable to Na_2_Ti_3_O_7_. The synthesis of the compound is straightforward and facile, implying easy scaling up, if required. Apparently, there is still a large room to improve and optimize the performance of this new compound as anode in NIBs, such as directly synthesizing this phase in nanostructures. High energy ball-milling can be used to reduce the particle size. But this can also introduce high-density defects in the particles, which may lower the performance. Well-crystalized nanostructures from direct synthesis may reduce the particle size without creating defects. This new crystal structure also provides opportunities for materials design to develop compounds with better performances. Structure and property tuning with doping of other metals should be considered in the future. For example, the poor electronic conductivity may be improved by doping with small amount of 3^+^ or 4^+^ metals such as Co or Ru. Doping with other metals or tuning the occupancy of Al at Ti site may help stabilize the end-of-discharge structure.

## Conclusions

In this work, a new sodium titanate was successfully synthesized and tested as the anode for sodium ion batteries for the first time. This material shows a very high first discharge capacity of ~225 mAh/g, yet with a large irreversible portion. The material shows very stable reversible capacity ~62 mAh/g in >100 cycles in half-cell setup. This material has a moderate rate capability with applying carbon coating. *In situ* XRD reveals that the material undergoes a solid solution-like phase evolution pathway with very small volume strain, which is advantageous for long-term cycling. More importantly, this compound provides a new structural motif other than Na_2_Ti_3_O_7_ and Na_2_Ti_6_O_13_. We believe there is a lot of room in compositional design and process optimization to further improve the performance of this compound and to design new compounds with similar structure and better performances.

## Methods

### Sample Synthesis

NaAlTi_3_O_8_ was synthesized through solid-state-reaction using stoichiometric amount of Na_2_CO_3_ (99.5% purity, Alfa Aesar), Al_2_O_3_ (99.8% purity, sigma-Aldrich) and anatase- TiO_2_ (>99.5% purity, Sigma-Aldrich). 0 wt %, 20 wt %, and 33 wt % sucrose (99% purity, Alfa Aesar) were added to the mixture to form carbon coated samples, denoted as 0 C, 22 C, 33 C hereafter, respectively. The mixture was ball-milled at 400 rpm for 1 hour using a Retsch PM200 Ball Mill, and pressed into pellets by MTI 12 T Lab Pressing YLJ-12. The pellets were calcined under argon flow in a Lindberg/Blue M tube furnace at 700 °C for 10 h or 700 °C -10 h followed by 950 °C - 2 h.

### Characterizations

A D8 Advance X-ray Diffractometer (Bruker AXS, Germany) with a Molybdenum radiation [λ Kα_1_ = 0.7093 Å] was used to examine the crystalline phase of as-sintered compound. LEO1550 Thermally-Assisted Field Emission (TFE) Scanning Electron Microscope(SEM) was used to characterize the particle size under different synthesis treatments. FEI Tecnai F30 Transmission Electron Spectroscope (TEM) was used to characterize the particle morphologies and conduct Energy Dispersive X-ray Spectroscopy (EDS) and Electron Energy-Loss Spectroscopy (EELS) analysis on particles under different synthesis conditions. Synchrotron X-ray diffraction was performed at beam line X14A at the National Synchrotron Light Source (NSLS) at Brookhaven National Laboratory (BNL) with using a linear position sensitive Si detector. The wavelength was 0.7692 Å. Synchrotron data was also collected at the beam line 17-BM at the Advanced Photon Source (APS) at Argonne National Laboratory for confirmation of the results and for comparison of different samples. Rietveld refinement was performed with using GSAS software^32^. Neutron scattering data was collected at the VULCAN beam line^33^ at the Spallation Neutron Source (SNS) at Oak Ridge National Laboratory (ORNL).

### Electrochemical testing

For electrochemical testing, composite electrodes were prepared. Carbon black (99+ % purity, Alfa Aesar) was ball-milled with the active material with a weight ratio of 15:85 at 600 rpm for 1 hour. Then the mixture, additional carbon black, and Polyvinylidene fluoride (PVDF) (Sigma-Aldrich) were blended with a weight ratio of 8:1:1, using N-Methyl-2-pyrrolidone (NMP) (J.T.Baker) as solvent. The slurry was then applied on aluminum foil and dried in an oven at 60 °C for 2 h. The dried film was pressed with using a roller-press (MTI Corp.) to densify the electrode. The film was then cut into circular discs of 15 mm diameter using a disc cutter (MTI Corp. MSK-T-07). Standard CR2016 coin cell was assembled by using sodium foil (99.95% purity metal basis, Sigma-Aldrich) as anode, NaAlTi_3_O_8_ composite electrode as cathode, 1 M NaPF_6_ (Sigma-Aldrich) in ethylene carbonate/diethylene carbonate (EC : DEC) as electrolyte, and Glass Microfiber Filter (VWR) as separator, in an argon filled glove box (MBraun). The cycling of coin cell was conducted with using a battery cycler (Arbin, BT2043), with voltage windows of 0.01 - 2.5 V or 0.1 - 2.5 V under rates of C/10, C/5, and 1 C. All electrochemical tests were performed under ambient temperature.

## Electronic supplementary material

Supplementary Information
