# Author Correction: NaAlTi_3_O_8_, A Novel Anode Material for Sodium Ion Battery

**DOI:** 10.1038/s41598-018-20583-y

**Published:** 2018-02-01

**Authors:** Xuetian Ma, Ke An, Jianming Bai, Hailong Chen

**Affiliations:** 1 The Woodruff School of Mechanical Engineering, Georgia Institute of Technology, Atlanta, GA 30332 USA; 2 Chemical and Engineering Materials Division, Oak Ridge National Laboratory, Oak Ridge, TN 37831 USA; 3 National Synchrotron Light Source II, Brookhaven National Laboratory, Upton, NY 11973 USA

Correction to: *Scientific Reports* 10.1038/s41598-017-00202-y, published online 13 March 2017

The original version of this Article contained a typographical error in the spelling of the author Jianming Bai, which was incorrectly given as Jianmin Bai. This has now been corrected in the PDF and HTML versions of the Article.

