# Supplementary material for: NaAlTi3O8, A Novel Anode Material for Sodium Ion Battery
Source: Sci Rep. 2017 Mar 13;7:162. doi: 10.1038/s41598-017-00202-y (PMC5427963; doi:10.1038/s41598-017-00202-y)
Supplement: Supplementary file 1 — Supplementary Information [file 41598_2017_202_MOESM1_ESM.pdf]

Supplementary Information

$NaAlTi_3O_8$: A Novel Anode Material for Sodium Ion Battery

Xuetian Ma[1], Ke An[2], Jianmin Bai[3], Hailong Chen[1*]
1. The Woodruff School of Mechanical Engineering, Georgia Institute of Technology, Atlanta, 30332, USA
2. Chemical and Engineering Materials Division, Oak Ridge National Laboratory, Oak Ridge, TN, 37831, USA
3. National Synchrotron Light Source II, Brookhaven National Laboratory, Upton, NY, 11973, USA
[*]Correspondence should be addressed to: hailong.chen@me.gatech.edu

1. **EDS mapping of 20 C-$NaAlTi_3O_8$ without ball-milling with carbon black**

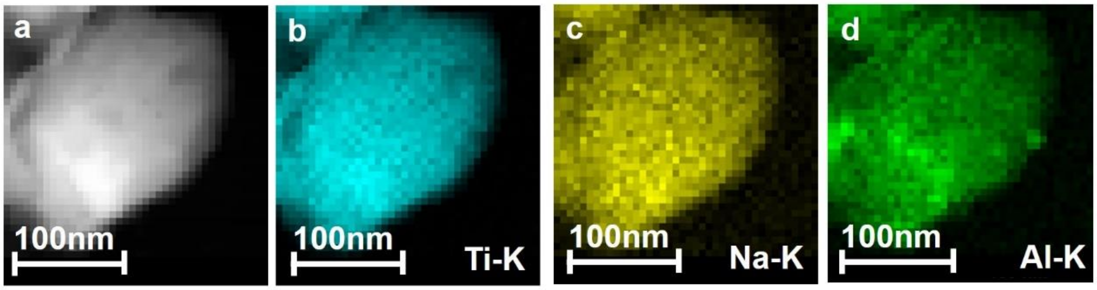

Figure S1. (a) STEM image of 20 C-NaAlTi3O8 without ball-milling with carbon black and EDS mapping of Ti-K(b), Na-K(c), and Al-K(d) from sample in (a).

2. **No carbon coating sample (0 C) electrochemical data**

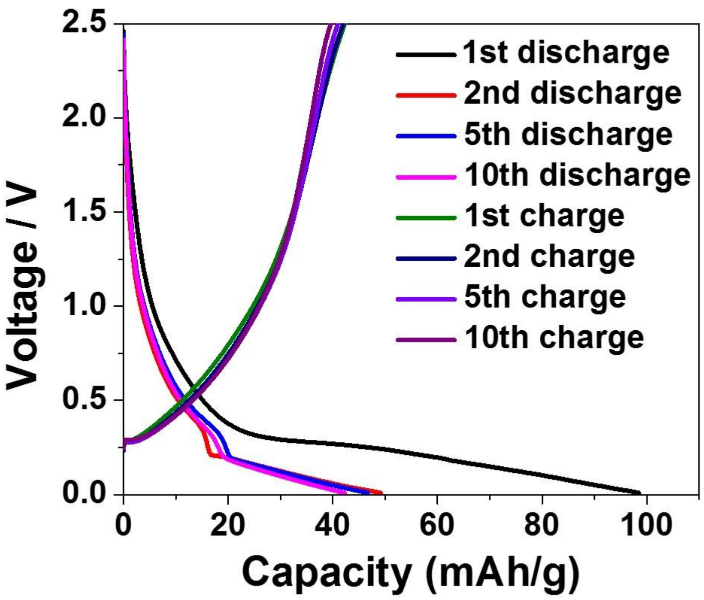

Figure S2. 1st, 2nd, 5th, and 10th discharge and charge profile at the rate of C/5 of 0 C sample under 700 °C for 10 h followed by 950 °C for another 2 h.

3. **Carbon black cell cycling data**

Figure S3 shows the electrochemical data for pure carbon cell cycled at C/10 rate between 2.5 V and 0.01 V. The anode of the carbon cell is sodium metal with pure carbon black

being the cathode, and 1M $NaPF_6$ (Sigma - Aldrich) in ethylene carbonate/diethylene carbonate (EC : DEC) as electrolyte.

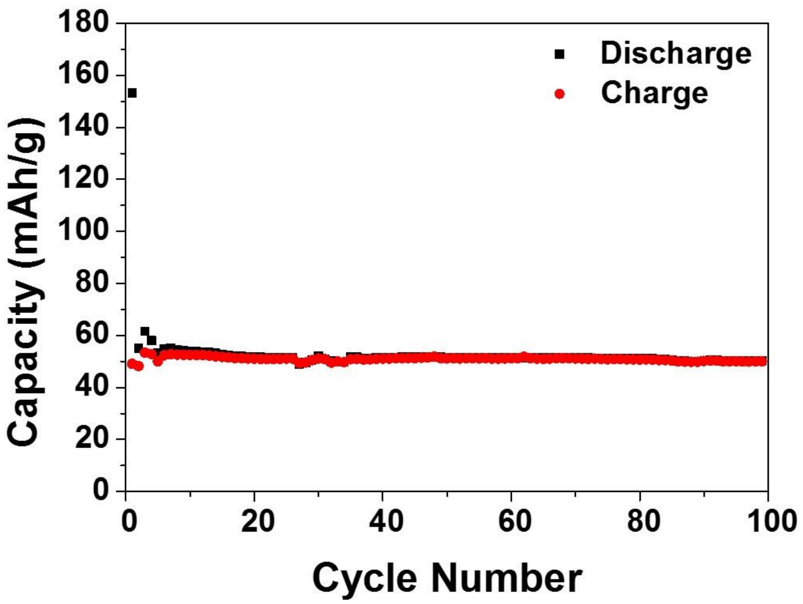

Figure S3. Electrochemical data of the cycling capability of pure carbon cell at C/10 rate between 2.5 V and 0.01 V.

4. *In situ* cell configuration

Figure S4 shows the configuration of the *in situ* cell for *in situ* XRD test. The cell has a similar yet modified configuration as in reference[1]. A beryllium window on top can allow X-ray to transmit through.

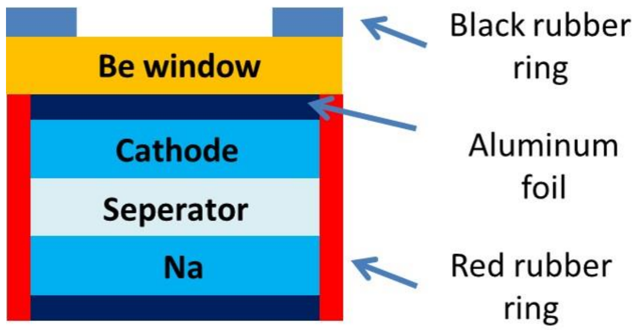

Figure S4. Configuration of *in situ* cell for *in situ* XRD test.

5. SEM images of 20 C sample before and after 20 cycles at C/10 with voltage window of 0.01 - 2.5 V.

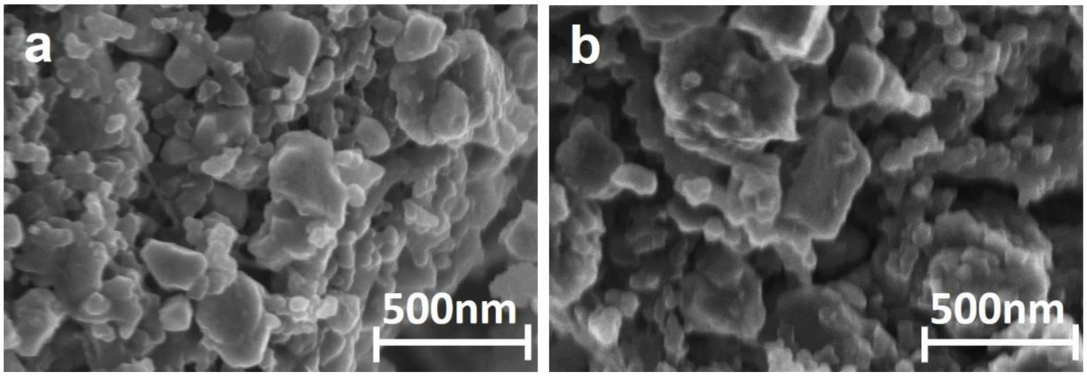

Figure S5. (a) SEM image of 20 C sample under 700 ℃ for 10 h and 950 ℃ for 2 h, followed by ball-milling with carbon black at 600 rpm (before cycle). (b) SEM image of the same sample after 20 cycles under C/10 rate between 0.01 - 2.5 V.

6. Ex situ XRD of 20 C sample cycled at C/10 after 20 cycles

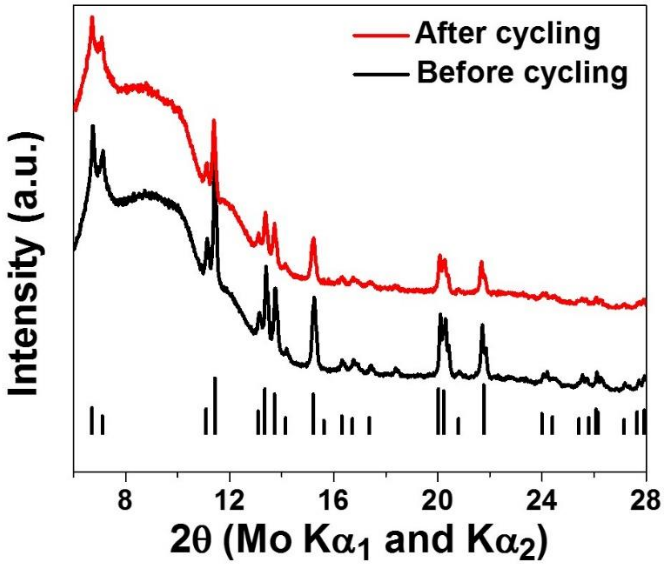

Figure S6. Ex situ XRD of 20 C sample before and after cycling at C/10 between 0.01-2.5 V after 20 cycles.

Reference

1. Wilson, B. E., Smyrl, W. H. & Stein, A. Design of a Low-Cost Electrochemical Cell for In Situ XRD Analysis of Electrode Materials. *J. Electrochem. Soc.* **161,** A700–A703 (2014).
